# Thiol Isomerases: Enzymatic Mechanisms, Models of Oxidation, and Antagonism by Galloylated Polyphenols

**DOI:** 10.3390/antiox14101193

**Published:** 2025-09-30

**Authors:** Osamede C. Owegie, Quinn P. Kennedy, Pavel Davizon-Castillo, Moua Yang

**Affiliations:** 1 Bloodworks Northwest Research Institute, Seattle, WA 98102, USA; oowegie@bloodworksnw.org (O.C.O.);; 2 Seattle Children’s Hospital, Seattle, WA 98102, USA; 3 Division of Hematology and Oncology, Department of Medicine, University of Washington School of Medicine, Seattle, WA 98102, USA

**Keywords:** thiol isomerase, disulfides, protein disulfide isomerase, polyphenol, thrombosis, hemostasis

## Abstract

Thiol isomerases are a family of enzymes that participate in oxidative protein folding. They contain highly reactive vicinal thiols in a CXXC motif within their catalytic domains to mediate thiol-disulfide switching as part of their reductase, oxidase, and isomerase activity. In addition, they participate in chaperone function by binding to partially folded or misfolded proteins and preventing aggregation, thereby facilitating correct protein folding. The CXXC motif is conducive to oxidative influence based on the sulfur nucleophilicity. Redox modification of the CXXC motif may influence the enzymatic function. In this review we briefly discuss the family of thiol isomerases as it relates to thrombotic disorders. We then discuss the chemical mechanisms of making and breaking disulfides by the enzymes. Enzymatic and chemical models of oxidizing the CXXC motif are proposed. Lastly, we highlight evidence that natural galloylated polyphenols can inhibit both the coronavirus main protease Mpro and thiol isomerases, supporting a therapeutic strategy for COVID-19-associated coagulopathy and thrombosis by targeting the CXXC motif with these anti-oxidative compounds.

## 1. Introduction

Christian Anfinsen’s pioneering work demonstrated that all the information required for a protein to attain its functional three-dimensional structure is encoded in its primary amino acid sequence. This landmark finding, often referred to as the thermodynamic hypothesis of protein folding, provided the conceptual framework showing that proteins can spontaneously fold in vitro into their native conformations. This contribution earned him the Nobel Prize in Chemistry in 1972. Within this broader context, the discovery of Protein Disulfide Isomerase (PDI) by Anfinsen and colleagues in 1964 further advanced the field by identifying an enzymatic catalyst capable of accelerating disulfide bond formation and rearrangement during oxidative protein folding [1,2]. This discovery laid the foundation for the identification of an entire family of thiol isomerases, characterized by conserved CXXC motifs and thioredoxin-like domains that define their redox activity [3,4]. These enzymes enable thiol-disulfide exchange reactions critical for maintaining protein stability and cellular homeostasis, and as such, are essential to life.

PDI was first identified in the endoplasmic reticulum (ER), where it plays a central role in oxidative protein folding [5]. By catalyzing disulfide bond formation and rearrangement, PDI ensures the structural integrity of secreted and membrane proteins, preventing misfolding and aggregation. Over subsequent decades, research expanded to reveal that PDI and its family members also exhibit diverse roles beyond oxidative folding, including chaperone activity and redox signaling [3,6,7]. These multifunctional enzymes act as molecular adaptors, responding to dynamic cellular conditions, such as ER stress, to maintain proteostasis [8,9].

From the 1990s, the focus on thiol isomerases shifted to their extracellular functions, particularly in thrombosis, hemostasis, and other vascular disorders. It was discovered that PDI is secreted by endothelial cells and platelets upon vascular injury, where it modulates key players in blood clotting, highlighting their potential as therapeutic targets for thrombotic disorders [10,11,12]. Despite their roles in thrombosis, the mechanism of escaping the confines of the ER is still being elucidated. Specifically, thiol isomerases were proposed to escape the ER through passive release by damaged or dead cells or by bypassing the Golgi apparatus during secretion [13]. In platelets and megakaryocytes, PDIs are packaged into noncanonical secretory granules (dense tubular system) for release into the extracellular environment where the KDEL ER-retention sequence is required for appropriate cellular transportation [14]. In recent years, the role of thiol isomerases in thrombosis has gained further attention due to their involvement in pathophysiologic conditions, such as obesity, diabetes, cardiomyopathy, cancer, and COVID-19-associated coagulopathy [15,16,17,18,19,20,21,22]. In these states, oxidative stress alters the redox state of their catalytic CXXC active site motif, shifting their balance between reductase and oxidase activities [23]. Reactive oxygen species (ROS) and reactive nitrogen species (RNS) oxidize the cysteines within the CXXC motif, promoting pathological disulfide bond formation in proteins important for thrombus formation [24]. Yet, significant knowledge gaps remain in understanding the molecular mechanisms by which thiol isomerases regulate disulfide bond dynamics under oxidative stress conditions.

The potential for targeting thiol isomerases as a therapeutic intervention is a research area of interest. Small-molecule inhibitors such as quercetin-3-rutinoside or rutin, and isoquercetin have demonstrated efficacy in preclinical mouse models of thrombosis by blocking PDI’s redox activity and reducing thrombus formation [25,26]. Isoquercetin, the derivative of rutin without the rhabinoside ring, has shown some promise by reducing platelet-dependent thrombin generation in pancreatic cancer patient plasma [27] and by decreasing thrombotic risk in patients without any reported bleeding events [28]. These studies are compared to historically matched control individuals [28]. Other small molecule antagonists, peptides, and blocking monoclonal antibodies have been discovered and reviewed elsewhere [11]. However, these compounds or antagonists to thiol isomerases potentially suffer from selectivity. Yet, potential inhibition of multiple thiol isomerases may present with selective advantages as it was proposed that thiol isomerase activity may be linked between members [24,29]. We recently found that the naturally occurring galloylated polyphenols, such as pinocembrin 7-O-(3″-galloyl-4″,6″-(S)-hexahydroxydiphenoyl)-β-D-glucose (PGHG) and punicalagin, are pan-thiol isomerase antagonists with antithrombotic effects. PGHG is a natural compound found in *Penthorum chinense* pursh, which is abundantly found in eastern Asia and used as a diet therapy for liver disease [30,31]. Found in pomegranate extracts, punicalagin broadly inhibited thiol isomerases (PDI, ERp57, ERp5, and ERp46) [32]. In mice, PGHG and punicalagin reduce thrombus formation without increasing bleeding in the tail transection model of hemostasis [32]. These compounds likely exert dual effects by suppressing thiol isomerase activity and alleviating oxidative stress, an important contributor to thrombotic disorders, through their antioxidative properties [32,33]. Despite these developments, critical questions remain regarding the mechanisms by which galloylated polyphenols interact with thiol isomerases. Further studies are needed to elucidate the mechanism of action affecting enzymatic activity under pathological conditions such as oxidative stress or inflammation. Additionally, optimizing these compounds for therapeutic use requires a deeper understanding of their specificity toward thiol isomerase while preserving physiological hemostasis.

In this review, we briefly discuss the structural diversity of the thiol isomerase family, deeply explore the chemical mechanisms by which thiol isomerases catalyze disulfide bond formation and cleavage and the oxidative modifications of the CXXC motif that regulate enzymatic function in pathological conditions. Finally, we highlight our findings on galloylated polyphenols as antithrombotic antagonists for thiol isomerases and discuss key unaddressed questions relating to the compounds.

## 2. Overview of Thiol Isomerase Family and Structural Diversity

The thiol isomerase family, also known as the PDI family, constitutes a diverse group of enzymes essential for oxidative protein folding, disulfide bond formation, and redox regulation [4]. These enzymes play critical roles in maintaining cellular homeostasis, particularly in the ER, where they assist in the maturation and stabilization of secretory and membrane proteins [34]. The family includes 23 members, each with unique structural and functional adaptations tailored to specific cellular roles [24,35,36]. Despite sharing a common structural framework characterized by thioredoxin-like domains and conserved CXXC motifs, these enzymes exhibit remarkable diversity in their domain organization, substrate specificity, and physiological functions [35,37].

At the core of thiol isomerase function is the highly conserved CXXC motif, where two cysteine residues are separated by two variable amino acids (e.g., CGHC). This motif is central to their redox activity, enabling catalysis of thiol-disulfide exchange reactions, including disulfide bond formation, reduction, and isomerization in substrate proteins [38,39,40]. The number and arrangement of thioredoxin-like domains vary significantly among family members, influencing their substrate specificity and functional versatility [41]. Many thiol isomerases also contain flexible X-linker regions that connect their domains, providing structural flexibility and enabling dynamic interactions with substrates [7,42]. Furthermore, post-translational modifications, including phosphorylation, can modulate the activity and localization of thiol isomerases, adding another layer of complexity to their regulation [43].

**Figure 1:**
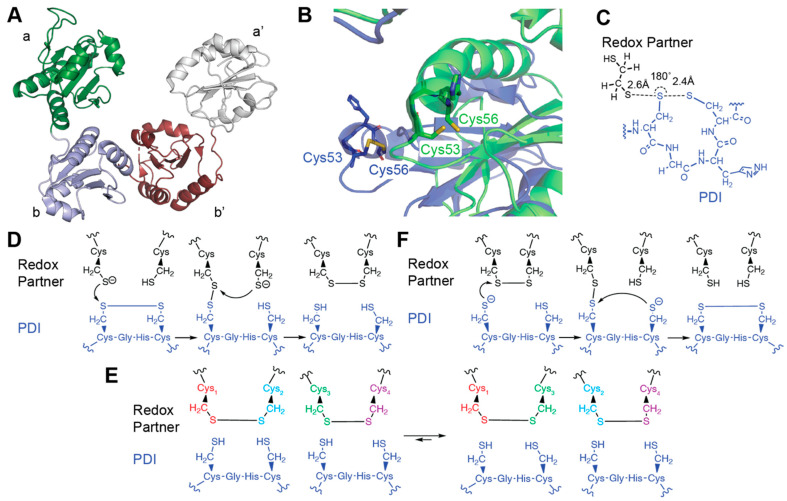
Protein Disulfide Isomerase (PDI) thioredoxin-like domain configuration and enzymatic activity. (**A**) The thioredoxin-like **a-b-b′-a′** domain configurations of PDI are shown. (PDB: 4EKZ). (**B**) Overlay of the CGHC catalytic motifs of the **a′** domains in the reduced (green; PDB: 4EKZ) or oxidized disulfide states (blue; PDB: 4EL1). (**C**) Catalysis requires a 180-degree orientation between the thiols of the CGHC catalytic motif in PDI and redox partner thiols. Figure adapted from [45] with permission. The oxidase activity (**D**), isomerase activity (**E**), and reductase activity (**F**) of PDI are shown. In (**E**) the colors represent distinct cysteine pairing after isomerization.

PDI, the prototypical member of the family, exemplifies the structural complexity of thiol isomerases. As a highly abundant thiol isomerase in the ER, PDI functions as a central catalyst of disulfide bond formation, reduction, and isomerization, ensuring proper folding of a wide range of newly synthesized proteins. Its activity is crucial for maintaining ER homeostasis and preventing the accumulation of misfolded proteins. PDI is composed of four thioredoxin-like domains arranged in a U-shaped structure: two catalytic domains (**a** and **a′**) containing the redox-active CXXC motifs (CGHC), and two non-catalytic domains (**b** and **b′**) that facilitate substrate binding and chaperone activity [39,44]. These thioredoxin-like domains are shown in Figure 1A. The **a** and **a′** domains are responsible for the enzyme’s ability to catalyze disulfide bond formation and rearrangement, while the **b** and **b′** domains contribute to substrate recognition and protein–protein interactions. A critical feature of PDI’s structure is the X-linker region, a flexible peptide sequence that connects the **b′** and **a′** domains. This linker region enhances the conformational flexibility of PDI, allowing it to adopt different orientations and interact with a wide range of substrates [7,39,42]. Upregulation of PDI expression and activity during ER stress

underscores its critical role in maintaining ER homeostasis and preventing the accumulation of misfolded proteins [9]. In addition, although PDI presumes a closed structural configuration in the reduced state compared to the open configuration in the oxidized state based on X-ray crystallography [40], atomic force microscopy [46], and single-molecule fluorescence resonance energy transfer (smFRET) studies [47,48] suggest a more dynamic structural configuration for PDI in specific redox states.

The structural diversity in the other thiol isomerase family members is shown in Table 1. This table represents the family members in human form [24] but includes the amino acid numbers of the CXXC motif. Some members have all four domains (e.g., PDI, ERp57). Other members have some, but not all, of the thioredoxin domain configurations as PDI (e.g., ERp5, ERp46). The structural diversity probably allows for differential function of the enzymes with defined substrates. In addition, although most of these enzymes are found within the ER, their presence extracellularly, on membrane surfaces, and in different tissues and organs may be the reason for diverse substrates and functions.

The diverse substrate specificity and range of interacting partners among thiol isomerase family members remain incompletely characterized, representing a significant gap in our understanding of their functional roles. In addition, the regulatory mechanisms governing the thiol isomerase family member expression, localization, and redox switching under physiologic or pathological conditions, such as ER stress or thrombosis, are also not well defined. It is unlikely that a single substrate mediates the functional effects of thiol isomerases but that multiple substrate-centric networks coordinate a net effect of thiol isomerases in reduced or oxidizing conditions [24]. 

### Function of Vascular Thiol Isomerases in Thrombotic Disorders

Vascular thiol isomerases are important in thrombosis through their multifaceted roles. They are secreted from platelets and endothelial cells and support platelet activation, fibrin formation, and thromboinflammation. These enzymes catalyze thiol-disulfide exchange reactions that modulate proteins involved in the blood clotting process. Thrombospondin secreted from vascular cells was one of the first substrates identified by secreted PDI [49,50]. Other substrates include platelet-derived proteins such as integrins [51,52,53,54,55,56] and Glycoprotein Ibα [57]. Endothelial cell-derived von Willebrand factor (vWF) [58] and vascular tissue factor (TF) [59,60,61] were shown to be potential substrates of thiol isomerases; however, regulation of vWF and TF by thiol isomerases in thrombus formation in vivo remains to be determined. Other proteins include coagulation proteins Factor Va [27], fibrinogen [62], and histidine-rich-glycoprotein [63]. Their involvement in thrombotic disorders is complex. To date, we do not know the network of substrates with disulfides reduced, cysteines oxidized, or disulfides isomerized for thrombus formation. We refer readers to these review articles on the current substrates of vascular thiol isomerases relevant to thrombosis [11,12,16,24,41,64,65].

## 3. Chemical Mechanisms of Thiol Isomerase Oxidoreductase Activity

Thiol isomerases, exemplified by PDI, catalyze thiol-disulfide exchange reactions fundamental to oxidative protein folding and extracellular redox signaling [6,66,67]. These enzymes mediate three distinct chemical activities—oxidation, reduction, and isomerization of disulfide bonds through a conserved catalytic mechanism governed by the dynamic reactivity of their CXXC active-site motif. In PDI, this motif is coded as a cysteine-glycine-histidine-cysteine (CGHC) that exists in a reduced or oxidized disulfide state [3,7,68] (shown in Figure 1B). The catalytic versatility of PDI arises from its modular domain organization comprising four thioredoxin-fold domains arranged in a twisted U-shaped conformation [69]. The **a** and **a′** domains housing the catalytic CXXC motifs share 37% sequence identity while functioning independently to perform disulfide-bond reduction, oxidation and isomerization [70]. The **b′** domain forms a hydrophobic substrate-binding cleft (residues Phe240–Leu294 in human PDI) that selectively binds unfolded polypeptide regions, while the c domain stabilizes interdomain interactions essential for complex substrate processing. Recent structural studies have revealed how conformational flexibility of hinge regions between these domains enables adaptive substrate recognition and efficient substrate handoff between domains [47].

At the molecular level, these reactions proceed through a bimolecular nucleophilic substitution (SN2) mechanism at sulfur centers, requiring strict linear alignment (180°) between the attacking thiolate, the central disulfide sulfur, and the leaving group to achieve optimal orbital overlap in the trigonal bipyramidal transition state [71]. This is shown in Figure 1C. The thioredoxin fold architecture enforces this geometric precision, utilizing backbone carbonyl groups to stabilize the developing negative charge through hydrogen bonding interactions [72]. The nucleophilic cysteine (Cys1) of the CXXC motif exhibits a remarkably low pKa (5.0–6.7) compared to free cysteine (~8.5), enabling thiolate formation under physiological conditions [73,74,75,76]. This pKa depression arises from multiple stabilizing factors, including alignment with the positive end of an α-helix macrodipole (~3.5 Debye), hydrogen bonding from backbone amides (NH···S^−^ distances ~3.4 Å), and charge-dipole interactions with neighboring histidine or lysine residues [75]. In the oxidized state (Cys1-S-S-Cys2), both cysteines are covalently linked as a disulfide and not protonated. However, these same microenvironmental stabilizing effects that depress the cysteine pKa in the reduced state also contribute to a highly oxidizing active-site disulfide, facilitating efficient thiol–disulfide exchange reactions [74,76,77,78]. The intervening residues in the CXXC motif have been widely described for their influence on the reduction potentials and subsequent disulfide stabilities, with variations in CXXC motif residues tuning redox potentials by up to 148 mV to allow functional specialization across cellular compartments [73,79,80].

### 3.1. Oxidase

In the oxidase cycle of thiol isomerases, disulfide bond formation begins with PDI in its oxidized state, typically containing a disulfide bond between the two cysteines of its CXXC active site motif (Figure 1D). This redox state is maintained by upstream oxidants, most notably Ero1 (endoplasmic reticulum oxidoreductin 1), which accepts electrons from reduced PDI and passes them to molecular oxygen via its FAD cofactor, thereby generating hydrogen peroxide [81,82]. The disulfide in the CXXC motif thus acts as an electrophilic sulfur acceptor, poised to transfer its oxidizing equivalents to a substrate bearing reduced thiols [83,84,85]. The reaction proceeds through an SN2-type displacement in which a substrate thiolate attacks the disulfide bond of the oxidized enzyme, forming a transient mixed disulfide intermediate and releasing the enzyme’s C-terminal cysteine (Cys2) as a thiolate. This transition state is governed by strict stereo-electronic constraints: the nucleophilic sulfur, central disulfide sulfur, and leaving sulfur must adopt a collinear 180° arrangement to permit optimal orbital overlap, forming a trigonal bipyramidal transition geometry. The active site of the enzyme stabilizes this state via hydrogen bonding from backbone amides and favorable electrostatic alignment from the α-helix dipole and adjacent charged residues, including conserved lysine and histidine residues that act as general bases to facilitate thiolate formation [86,87].

The formation of the substrate thiolate nucleophile is itself a rate-limiting event, particularly under physiological pH, as the unmodified pKa of a cysteine thiol is approximately 8.5, rendering it largely protonated and less nucleophilic at neutral pH [72]. However, the local protein environment can modulate this value through proximity to positively charged residues, and through desolvation effects that stabilize the conjugate base. In some systems, enzyme-bound water molecules act as proton shuttles, relaying the proton from the substrate thiol to a general base such as His55 in human PDI, which typically has a pKa around 6.5 and is ideally positioned to perform proton abstraction.

Once the substrate thiolate has formed and attacked the enzyme disulfide, a mixed disulfide intermediate (E–S–S–R) is produced, and the resolution of this intermediate becomes the subsequent and often rate-determining step. In this step, a second cysteine within the same substrate protein, which must be appropriately folded to be in spatial proximity, performs an intramolecular nucleophilic attack on the mixed disulfide bond. This step releases the enzyme in its reduced form and generates a disulfide bond within the substrate. The resolution again proceeds via a trigonal bipyramidal transition state, requiring proper geometric alignment. The ability of the substrate cysteine to perform this attack depends on its thiol pKa and spatial orientation. The enzyme facilitates this step through its substrate-binding domains (especially the **b′** domain in PDI), which align the substrate in a catalytically competent conformation [88]. Structural studies have shown that the **b′** domain forms a hydrophobic pocket that selectively binds unfolded or partially folded polypeptide regions, increasing the local effective concentration of reactive thiols [88].

Moreover, conformational flexibility within the active site loop that houses the CXXC motif plays a critical role. This loop must transition between an open conformation that permits substrate access and a closed conformation that promotes disulfide exchange while excluding solvent and preventing hydrolysis. NMR and crystallographic analyses indicate that loop dynamics are finely tuned in response to substrate binding, redox state, and the presence of electron transfer partners [76]. In human PDI, residues surrounding the CGHC motif modulate the accessibility and reactivity of the active site; substitutions within this loop significantly impair catalytic rates, underscoring its allosteric function. During catalysis, the loop folds over the active site to form a tight microenvironment that stabilizes negative charge buildup on the leaving thiolate and supports optimal orbital alignment for the SN2 transition state.

Following disulfide bond formation in the substrate, the enzyme is left in a reduced state with both active-site cysteines as free thiols. In order to complete the catalytic cycle and continue functioning as an oxidase, the reduced enzyme must itself be reoxidized. This is achieved via intramolecular electron transfer from one catalytic domain to another (for example, from **a′** to **a** in PDI), or through direct interaction with an ER oxidase such as Ero1, PRDX4, or vitamin K epoxide reductase (VKOR). Ero1, in particular, forms transient mixed disulfides with PDI and transfers oxidizing equivalents via its FAD cofactor, which then reduces molecular oxygen to H_2_O_2_ [89]. These oxidative partners typically display a redox potential more positive than that of the enzyme (~−150 mV for Ero1 versus ~−180 mV for PDI), ensuring favorable electron flow because electrons flow from the more electronegative to the more electropositive redox potential [23,29,90].

Oxidase activity is tightly coupled to the ER oxidative machinery, particularly Ero1α/β and PRDX4, which reoxidize PDI’s **a** domains [82]. The **a** domain disulfide (Cys53–Cys56 in human PDI) acts as the primary electron acceptor, with its high redox potential (−175 mV) favoring disulfide transfer to substrates [91]. The **b′** domain facilitates oxidase function by binding unfolded proteins, increasing the local concentration of substrate thiols near the catalytic **a** domain [88].

Notably, the **a′** domain shows attenuated oxidase activity due to its more negative redox potential, but it becomes critical for oxidizing large, multidomain substrates (e.g., thrombospondin-1 [50]) through collaborative interactions with the **a** domain [68]. The **c** domain (C-terminal extension) further regulates oxidase activity by interacting with Ero1, as truncation experiments show a 60% reduction in disulfide transfer efficiency [92]. Kinetic studies indicate that oxidase rates are 3–5-fold slower than reductase rates, reflecting the energetic cost of de novo disulfide formation [81,82].

### 3.2. Isomerase

The isomerase activity of thiol isomerases underlies their role in correcting non-native disulfide bonds in misfolded proteins, making it arguably their most complex chemical function [68]. The isomerase mechanism is conceptually a composite of reductase and oxidase activities: the enzyme must first reduce an incorrect disulfide bond and then oxidize two appropriate cysteine residues to form a new, native disulfide [68,93]. This necessitates not only bond reshuffling but also protein conformational rearrangements that allow the correct cysteines to come into proximity. Historically, the isomerase activity is what gave thiol isomerases the “disulfide shufflase” moniker for protein folding. To date, disulfide shufflase is no longer a standard moniker for the isomerase activity.

The isomerase reaction begins with the reduced form of PDI (Cys1–S^−^, Cys2–SH), which initiates attack on a non-native disulfide bond. As in the reductase mechanism, this leads to formation of an enzyme–substrate mixed disulfide. The resolution of this intermediate state proceeds through the same steps as the deprotonation described above and nucleophilic attack by Cys2 to liberate a reduced substrate and regenerate the enzyme disulfide (Figure 1E).

However, the key distinction in isomerase function is that this reduced substrate is not released [94]. Instead, it remains transiently associated with the enzyme, particularly via hydrophobic contacts in domains such as the **b′** domain in PDI, which binds exposed unfolded regions of the substrate. This close association allows the enzyme to selectively oxidize the correct cysteine pairs within the same polypeptide. The second phase of catalysis thus involves oxidase activity of the now reoxidized enzyme disulfide, transferring the disulfide to the appropriate thiol pair to generate the correct bond [95].

Kinetically, the rate-limiting step in isomerization is typically the conformational rearrangement of the substrate between reduction and reoxidation. This rearrangement is often slow, taking place over milliseconds, and is highly dependent on the substrate’s folding landscape. Moreover, substrate affinity varies between PDI family members due to electrostatic and hydrophobic complementarity. For example, ERp57, a PDI family member, exemplifies how divergent domain architectures tailor isomerase specificity. Its **a** domains share the CGHC motif with PDI, but its **b′** domain preferentially binds glycoproteins via its interaction with calnexin/calreticulin, while PDI’s **b′** domain accommodates a broader range of substrates via a hydrophobic groove [96,97].

Thermodynamically, the isomerase activity is driven by the redox potential gradient between incorrect and correct disulfide bonds. Correct disulfide pairings are typically more stable, with lower Gibbs free energy. The enzyme ensures fidelity by maintaining a redox potential poised just oxidizing enough (~−180 to −200 mV) to allow selective formation of the correct disulfide without becoming kinetically trapped in partially reduced intermediates [68]. This tuning is supported by a precise balance of local pKa values, substrate-binding affinity, and domain-domain electron transfer kinetics.

Isomerization requires coordinated action of the **a** and **a′** domains for disulfide shuffling and the **b′** domain for substrate retention. The **b′** domain’s hydrophobic pocket (residues Phe240–Leu294 in human PDI) selectively binds misfolded polypeptides, holding them transiently after reduction to allow conformational sampling [97]. The **a** domain initiates attack on non-native disulfides, while the **a′** domain preferentially reoxidizes native cysteine pairs, leveraging its lower redox potential to avoid kinetic traps [76]. The **c** domain enhances isomerase efficiency by stabilizing interdomain interactions; truncated forms of the **c** domain exhibit a 70% loss in activity for complex substrates like RNase A [40].

### 3.3. Reductase

In the reductase cycle, the enzyme initiates catalysis from its reduced state (Cys1-S^−^/Cys2-SH) (Figure 1F). The nucleophilic thiolate attacks substrate disulfides with strict adherence to SN2 geometry, requiring perfect 180° alignment between the attacking sulfur, target disulfide, and leaving group. This geometric precision is facilitated by an oxyanion hole (residues 50–53) that stabilizes the transition state [45]. Substrate binding induces strain in the target disulfide, lengthening the S-S bond from 2.05 Å (ground state) to ~2.15 Å (transition state). Kinetic analyses show rate constants of 10^3^–10^4^ M^−1^s^−1^ and activation energies of 50–60 kJ/mol, with kinetic isotope effects (kH/kD ≈2–3) confirming proton transfer contributes to rate limitation [71]. The resolution of the enzyme–substrate mixed disulfide intermediate formed after the initial nucleophilic attack by the enzyme’s *N*-terminal thiolate (Cys1-S^−^) on a substrate disulfide is frequently the rate-determining step in the reductase cycle. In this intermediate, the enzyme’s Cys1 is covalently linked via a disulfide bond to one cysteine of the substrate, while the second catalytic cysteine (Cys2) remains in its protonated thiol form [98]. For catalysis to proceed, Cys2 must be deprotonated to generate a thiolate that can nucleophilically attack the adjacent sulfur atom, resolving the mixed disulfide and releasing the fully reduced substrate [98,99]. However, this step is thermodynamically challenging since Cys2 typically has a much higher intrinsic pKa (~10.5), making thiolate formation under physiological pH (~7.4) inefficient [76,87]. The enzyme mitigates this barrier through a constellation of local interactions including backbone strain in the CXXC loop that perturbs the pKa of Cys2 by ~1–2 units, destabilizing the thiol ground state and making deprotonation more favorable, and a conserved histidine residue immediately adjacent to Cys2 in the CGHC motif that often functions as a general base catalyst with its imidazole side chain (pKa ~6.5) abstracting the proton from Cys2-SH to enable thiolate formation in a concerted proton-coupled electron transfer event [3]. Once deprotonated, Cys2-S^−^ attacks the mixed disulfide from the enzyme side in another SN2-like displacement, cleaving the Cys1-substrate bond with precise 180° alignment between the attacking sulfur (Cys2), the central sulfur of the disulfide bond (Cys1), and the departing substrate sulfur [71]. The transition state is stabilized by hydrogen bonding networks and electrostatic interactions with helix macrodipoles, while aromatic side chains near the active site participate in π-sulfur interactions that stabilize charge buildup on the departing sulfur [75,100]. The net result is restoration of the enzyme to its oxidized state and release of the reduced substrate [68].

The **b′** domain plays a crucial role in positioning substrates for reduction through its hydrophobic cleft, and recent studies demonstrate that mutations in this domain reduce RNase A processing efficiency [88,101,102]. The **a** and **a′** domains exhibit functional redundancy in reductase activity, though the **a′** domain shows higher reductase activity due to its more negative redox potential (−195 mV vs. −175 mV for the **a** domain) [76,103]. The **b** domain, though lacking catalytic cysteines, contributes to reductase activity by modulating interdomain flexibility, ensuring optimal alignment of the **a** and **a′** domains with substrates [7,97].

While this catalytic process is central to normal protein folding, PDI’s reductase activity also plays a vital role in protein quality control, particularly during endoplasmic reticulum-associated degradation (ERAD) [104]. In ERAD, misfolded or aberrant proteins often need to have their disulfide bonds reduced prior to retrotranslocation to the cytosol for degradation by the proteasome [105]. PDI and select family members such as ERdj5 that act as reductases in this pathway, catalyzing the breaking of disulfide bonds that otherwise stabilize misfolded proteins and impede their clearance [106]. For example, PDI’s reductase activity is essential for the retrotranslocation and ERAD of mutant proinsulin and other misfolded secretory proteins, where it primes substrates by cleaving aberrant disulfides and enabling their extraction from the ER [105,106].

### 3.4. Enzymatic Oxidation of Thiol Isomerases

#### 3.4.1. The Endoplasmic Reticulum Oxidoreductin 1 (ERO1)

Endoplasmic Reticulum Oxidoreductase 1 (ERO1) is a flavoprotein oxidase that resides in the endoplasmic reticulum (ER) and is essential for oxidative protein folding in eukaryotic cells. ERO1 exists in two isoforms, ERO1α and ERO1β, in humans and other vertebrates [107,108]. In the ER, both ERO1α and ERO1β act as principal oxidases responsible for generating disulfide bonds in nascent polypeptides. They accomplish this by oxidizing PDI, which then transfers disulfide bonds to substrate proteins [89,107]. Among these isoforms, ERO1α serves as the primary enzyme that restores oxidized PDI through highly regulated redox relay mechanisms. ERO1β complements ERO1α by providing additional oxidizing capacity under conditions such as increased protein folding demand or ER stress, though it is often less tightly regulated than ERO1α [89,107]. Together, these two isoforms ensure robust and dynamic control over PDI oxidation, supporting efficient oxidative protein folding in the ER.

**Figure 2:**
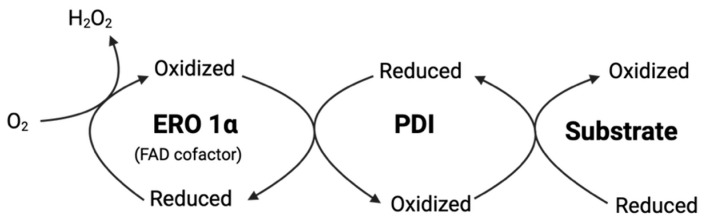
Mechanism of PDI oxidation by ERO1α. Oxidized PDI in the endoplasmic reticulum oxidizes and introduces disulfides to reduce protein substrates, becoming reduced in the process. ERO1α uses flavin adenine dinucleotide (FAD) as a cofactor to accept electrons from reduced PDI, resulting in ERO1α being reduced and PDI re-oxidized. Reduced ERO1α becomes re-oxidized by transferring electrons to diatomic oxygen, promoting hydrogen peroxide (H_2_O_2_) generation.

ERO1 operates through a FAD-dependent redox relay system involving two critical disulfide pairs: a catalytic disulfide (Cys94-Cys99 in human ERO1α) and a regulatory disulfide (Cys131-Cys134) that controls enzyme activity [91,107]. The mechanism of ERO1-mediated PDI oxidation is shown in Figure 2. Recent structural and mechanistic studies have revealed that both human ERO1 isoforms exist in dynamic mixed disulfide complexes with PDI, establishing a more complex regulatory framework than previously appreciated [108]. The oxidation mechanism proceeds through precisely coordinated thiol-disulfide exchange reactions wherein reduced PDI, with its active site cysteines in the thiol state (Cys53-SH/Cys56-SH), nucleophilically attacks the Cys94-Cys99 disulfide bond of ERO1, resulting in formation of a transient mixed disulfide intermediate between PDI and ERO1 (PDI-S-S-ERO1). The reaction progresses as ERO1’s Cys99 thiolate resolves this intermediate, ultimately releasing fully oxidized PDI (containing the Cys53-S-S-Cys56 disulfide) and reduced ERO1, with electrons abstracted during this process transferred through the FAD cofactor to molecular oxygen, generating hydrogen peroxide as a byproduct [91].

The H_2_O_2_ produced in this process is suggested to play a substantial role in the local concentrations of H_2_O_2_ in the ER, which can modulate redox signaling and serve as substrates for peroxidases such as peroxiredoxin IV (PRDX4) and glutathione peroxidases, further influencing the thiol-disulfide balance and limiting oxidative stress [91,109]. Recent docking simulations and systematic biochemical analyses have revealed that a protruding β-hairpin of ERO1α specifically interacts with the hydrophobic pocket present in the redox-inactive PDI **b′**-domain through aromatic residue stacking, leading to preferred oxidation of the C-terminal PDI **a′**-domain [85,110,111]. This electron transfer cascade is thermodynamically favorable due to the redox potential gradient between the participating molecules, with ERO1 maintaining a more oxidizing redox potential (−150 mV) compared to PDI (−180 to −200 mV), ensuring unidirectional electron flow [110,111]. The regulatory disulfides in ERO1 serve as a critical safety mechanism, undergoing reversible oxidation to prevent excessive ER hyperoxidation. PDI binding induces conformational changes in the ERO1α regulatory loop, promoting disulfide rearrangement and stabilizing the active Ox1 form relative to the inactive Ox2 form [108,110].

#### 3.4.2. Quiescin Sulfhydryl Oxidase

Quiescin sulfhydryl oxidase (QSOX) enzymes catalyze the formation of disulfide bonds de novo in unfolded proteins within the secretory pathway and extracellular environment, acting independently rather than directly forming the disulfide in the PDI active site [112,113]. QSOX1 does not contribute to disulfide bond formation in PDI’s active site, distinguishing it from classical ER oxidants such as ERO1. Instead, its physiological roles include introducing disulfide bonds into select glycosyltransferases in the Golgi and supporting extracellular matrix assembly, such as enhancing laminin incorporation and tissue remodeling [112,113,114]. While QSOX1 efficiently installs disulfide bonds, subsequent isomerization and proofreading rely on PDI and related isomerases. Under typical ER conditions, QSOX1 is less efficient than ERO1 and is primarily significant in compartments beyond the ER or under circumstances where ERO1 activity is limited [112,113,114]. QSOX enzymes are distinguished by their modular architecture, which merges an *N*-terminal thioredoxin-fold (Trx) domain containing a catalytic CXXC motif with a C-terminal Erv/Erv2 domain possessing a tightly bound flavin adenine dinucleotide (FAD) as a cofactor [115,116].

The oxidation mechanism of QSOX is a stepwise thiol–disulfide relay. Initially, reduced substrate protein thiols react with the *N*-terminal Trx CXXC motif of QSOX, resulting in the formation of a disulfide bond in the substrate and reduction of the Trx motif. Electrons are then relayed intramolecularly to the Erv/Erv2 domain. Here, the FAD cofactor plays a central and chemically direct role in oxidizing the intermediary cysteines. The redox-active center of FAD is the isoalloxazine ring, which undergoes two-electron chemistry. Specifically, the reduced cysteine disulfide in the Erv/Erv2 domain transfers electrons directly to the isoalloxazine ring of FAD, reducing it to FADH_2_ [117,118].

**Figure 3:**
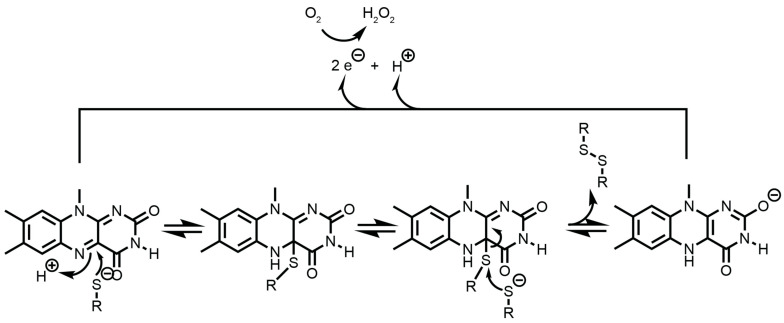
The mechanism of disulfide formation from transferring electrons to the isoalloxazine ring of FAD. Adapted from [119].

Once FAD accepts electrons and becomes reduced, it is rapidly reoxidized by molecular oxygen. The isoalloxazine ring of FAD donates two electrons to O_2_, regenerating oxidized FAD and yielding hydrogen peroxide (H_2_O_2_) as a byproduct. The direct involvement of the isoalloxazine ring is critical, crystallographic and mechanistic studies demonstrate that substrate electrons pass from protein thiols to the FAD isoalloxazine ring via transient mixed disulfide and internal relay intermediates, enabling efficient oxidation of unfolded proteins at turnover rates exceeding 700 disulfides per QSOX molecule per minute [116,117]. This mechanism is shown in Figure 3.

This mechanistic relay is unique among cellular oxidases. The isoalloxazine ring’s ability to stabilize both one- and two-electron redox transitions ensures that QSOX can catalyze the complete transfer of oxidizing power from O_2_ to protein dithiols without aberrant production of radical intermediates. Functional studies further reinforce that QSOX does not directly isomerize non-native disulfides; instead, it installs disulfides de novo, with subsequent isomerization or proofreading requiring PDI or related isomerases [115,116].

In the extracellular matrix, the H_2_O_2_ generated by isoalloxazine-mediated O_2_ reduction can further oxidize secreted thiol isomerases such as ERp5 and ERp46, amplifying redox signaling in processes like integrin activation and tissue remodeling [120].

In summary, QSOX is distinguished by its incorporation of the isoalloxazine ring of FAD as a direct redox center for thiol oxidation. This configuration enables a seamless and highly efficient transfer of electrons from protein thiols to molecular oxygen, producing disulfide bonds in substrate proteins and hydrogen peroxide as an oxidant for additional pathways.

#### 3.4.3. Glutathione Peroxidases

Glutathione peroxidases (GPx) are a family of antioxidant enzymes that protect cells from oxidative damage by catalyzing the reduction of hydrogen peroxide and organic hydroperoxides to water or alcohols, using glutathione as a reducing agent [121,122]. GPx enzymes play a central role in maintaining redox balance and cellular homeostasis, with several isoforms found in mammals, including GPx1–GPx8. The mechanism involves glutathione donating electrons to the peroxides, converting harmful oxidants to harmless products and oxidized glutathione, which is then recycled by glutathione reductase [121]. Recent studies have established that glutathione peroxidases 7 and 8 (GPx7 and GPx8) play a pivotal role as H_2_O_2_-dependent oxidants of protein disulfide isomerase (PDI) in the mammalian endoplasmic reticulum [122,123,124,125]. GPx7 is a highly efficient facilitator of PDI oxidation in the endoplasmic reticulum, directly using H_2_O_2_ as an electron acceptor to form the disulfide bond in the PDI active site [122,126]. Its activity is crucial for maintaining robust oxidative protein folding, especially under conditions where peroxide production outpaces ERO1 capacity or as part of an integrated redox relay involving both ERO1 and GPx7 [121,126]. Compared to GPx8, GPx7 is substantially more reactive and a significantly more effective source of oxidative equivalents for PDI, making it a physiologically important backup or parallel oxidant for PDI in mammalian cells [126,127]. The mechanistic origins of this disparity were elucidated through a combination of mutational, kinetic, and structural analyses, which highlighted the importance of the local active site environment in substrate specificity and catalytic proficiency [122,123,124,125]

**Figure 4:**
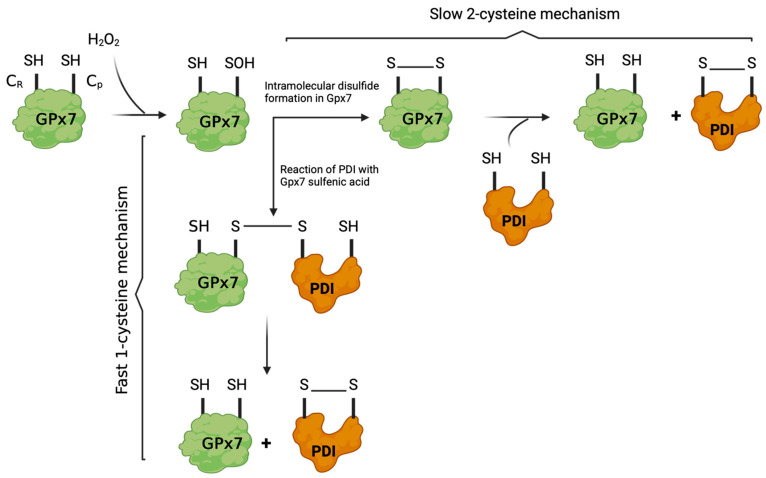
Two differential pathways for PDI oxidation by glutathione peroxidase 7 (GPx7). GPx7 has a peroxidatic cysteine (C_P_) and a resolving cysteine (C_R_). Reduced GPx7 becomes oxidized by H_2_O_2_ at the C_P_ forming a sulfenic acid (SOH) intermediate. At this stage, reduction of the sulfenic acid is mediated by competition between the C_R_ and reduced PDI. In a fast 1-cysteine mechanism, the nucleophilic cysteine in reduced PDI attacks the SOH on GPx7 forming a mixed GPx7-PDI intermediate prior to PDI’s vicinal thiol releasing GPx7. The release of the substrate renders PDI oxidized and GPx7 reduced. In the slow 2-cysteine mechanism, sulfenylated GPx7 becomes oxidized by the C_R_ to form a disulfide. Reduced PDI breaks the disulfide through its reductase activity to form oxidized PDI and reduced GPx7. Adapted from [123].

The mechanism of PDI oxidation by GPx7 is shown in Figure 4. The oxidation of reduced PDI by GPx7 in the presence of H_2_O_2_ proceeds via two distinct pathways, whose prevalence and kinetics are dictated by the available cysteine residues and their spatial arrangement within the peroxidase [123,127]. In the first and more rapid pathway, often designated the one-cysteine mechanism, H_2_O_2_ directly oxidizes the peroxidatic cysteine (Cys57 in human GPx7) to a sulfenic acid (Cys–SOH) intermediate [121,123,127]. This destabilized sulfenic acid is immediately trapped by an attacking cysteine in reduced PDI, generating a GPx7–PDI mixed disulfide. This intermediate then rapidly resolves to yield oxidized PDI (PDI with an active-site disulfide bond) and reduced GPx7 [123,127]. Kinetic analyses reveal that this one-cysteine relay is markedly faster, permitting GPx7 to achieve robust oxidation of PDI and efficient support of ER protein folding under physiological H_2_O_2_ loads [123].

In contrast, the second, slower pathway is known as the two-cysteine mechanism. Here, the initial step is identical: H_2_O_2_ oxidizes the peroxidatic cysteine to a sulfenic acid. Rather than reacting immediately with PDI, however, this sulfenic acid condenses with the resolving cysteine (Cr) within GPx7 itself to form an intramolecular disulfide bond [121]. In the subsequent step, a cysteine of reduced PDI attacks this internal GPx7 disulfide, eventually yielding oxidized PDI and further regenerating the reduced peroxidase for a new catalytic cycle [121,123]. Notably, while GPx7 can utilize both transfer mechanisms, GPx8, by virtue of its active-site architecture (determined primarily by Ser114 instead of Gln92), is reliant exclusively upon this two-cysteine mechanism. Consequently, GPx8 exhibits a considerably slower rate of PDI oxidation, a functional distinction that can be reversed in part by residue swapping at this key position [123].

Mutagenesis studies provided compelling evidence: substituting Gln92 of GPx7 for a serine (thus mimicking GPx8) drastically compromises GPx7’s peroxidatic capacity and PDI-oxidizing activity, essentially converting it into a GPx8-like, two-cysteine enzyme [123]. Conversely, the reciprocal substitution in GPx8 increases its hydrogen peroxide reactivity and confers partial competence for the one-cysteine, fast transfer mode. Crystallographic and biochemical data suggest that the size and polar nature of Gln92 help stabilize the transition state and facilitate the release of sulfenylated cysteine toward intermolecular thiol-disulfide exchange with PDI, rather than allowing premature internal resolution [123].

This system not only determines the fundamental kinetics of disulfide bond formation in the mammalian ER but also highlights an evolutionary adaptation in which GPx7 is tailored to be a highly specialized, efficient oxidase for PDI under oxidative stress or when high folding throughput is required. In practical terms, physiological demands for rapid and robust disulfide introduction into newly synthesized proteins are met by this dual pathway, with the one-cysteine mechanism functioning as a rapid relay particularly suited for intense secretory activity or stress responses, and the two-cysteine mechanism providing a slower, backup means for oxidative folding. The two-cysteine mechanism, typical for GPx8, may serve functions where the oxidative flux is lower or more tightly regulated. Together, these mechanistic pathways ensure that the ER retains both flexibility and robustness in its oxidative folding machinery, a capacity further fine-tuned by the relative expression and post-translational regulation of the peroxidases themselves.

The GPx7 pathway has been characterized extensively in vitro and in cell-based studies, with key mechanistic steps delineated through purified protein assays and cellular models [123,127,128]. Several publications confirm the reactivity and mechanism of GPx7-mediated PDI oxidation in mammalian cells, and in vivo evidence shows GPx7 participation in redox regulation and protein folding, but most detailed mechanistic insight comes from in vitro biochemical and cell culture systems. Full physiological relevance continues to evolve, but its functional importance in the ER is well-supported by experimental data [123,127,128].

#### 3.4.4. Peroxiredoxin 4

Peroxiredoxin 4 (PRDX4) is the sole member of the peroxiredoxin family localized within the endoplasmic reticulum (ER), where it has emerged as a central mediator of H_2_O_2_-dependent protein oxidation and a critical regulator of the redox environment supporting oxidative protein folding. It is a highly efficient oxidant of PDI in the mammalian ER, facilitating rapid oxidation of PDI and other family members through a thiol–disulfide exchange mechanism that transfers oxidizing equivalents derived from H_2_O_2_ and is often more effective than ERO1, particularly under conditions of increased oxidative folding demand or when H_2_O_2_ is abundant [129]. Its activity ensures that disulfide bond formation can proceed efficiently and robustly via an alternative pathway, enhancing both the rate and fidelity of oxidative protein folding in the secretory pathway [129,130].

**Figure 5:**
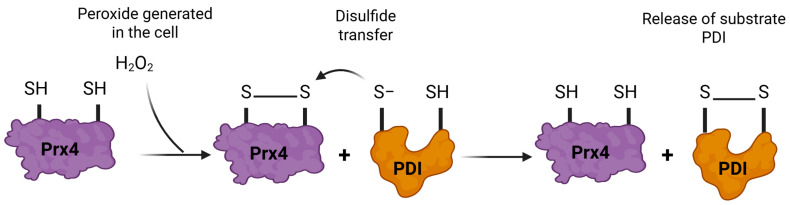
Mechanism of PDI oxidation by peroxiredoxin 4 (Prx4). Reduced Prx4 is oxidized by H_2_O_2_ in the cell forming disulfides on the enzyme. PDI then reduces the disulfided Prx4, allowing for the transfer of disulfides to PDI.

Mechanistically, PRDX4 functions as a typical 2-Cys peroxiredoxin. The mechanism is shown in Figure 5. Upon encountering H_2_O_2_, the highly conserved peroxidatic cysteine (Cp, Cys124 in human PRDX4) is oxidized to a sulfenic acid (Cys–SOH). This sulfenic acid then forms an intermolecular disulfide bond with the resolving cysteine (Cr, Cys245) of another PRDX4 subunit, yielding a disulfide-linked PRDX4 dimer; this is the “peroxidase cycle” seen in other peroxiredoxins [109,129]. In the ER, the oxidized dimeric PRDX4 is reduced by substrate proteins such as PDI, ERp46, or ERp5 via a thiol–disulfide exchange: the nucleophilic cysteine in the substrate attacks the PRDX4 disulfide, forming a transient mixed disulfide intermediate before resolving to release oxidized substrate (e.g., PDI with a disulfide in its CXXC motif) and regenerate reduced PRDX4 [130,131].

This redox relay enables PRDX4 to efficiently transfer oxidizing equivalents from H_2_O_2_ to a variety of ER-localized thiol isomerases and folding enzymes. Notably, PRDX4 recognizes and preferentially oxidizes ERp5 and ERp46, with the PRDX4-mediated oxidation of these proteins being markedly accelerated in the presence of PDI. This suggests PRDX4 and PDI do not act in isolation, but operate in a coordinated or even hierarchical network with substrate channeling among ER oxidases, thereby amplifying the efficiency and selectivity of disulfide bond formation during protein folding [16,111,131].

Under conditions of oxidative overload, excessive peroxide (such as tert-butyl hydroperoxide) can hyperoxidize the peroxidatic cysteine of PRDX4, converting it first to a sulfinic acid (SO_2_H) and then to an irreversible sulfonic acid (SO_3_H). This modification disrupts the disulfide relay, induces PRDX4 oligomerization into high-molecular-weight complexes, and results in a functional switch from peroxidase to chaperone activity. This oxidative transition is not only accompanied by changes in PRDX4’s structure and assembly but also by the recruitment of partner ER proteins, including PDI and other protein disulfide isomerases, into supramolecular complexes that help buffer protein folding stress and maintain proteostasis [129].

Collectively, PRDX4 provides a pivotal pathway for coupling ER-localized H_2_O_2_ production to the enzymatic oxidation of PDI family proteins via a well-defined, multi-step redox relay. It ensures the efficient use of peroxide for protein oxidation under physiological conditions while employing dynamic structural mechanisms to mitigate damage and preserve cellular homeostasis under oxidative stress. This exemplifies the adaptive strategies by which ER peroxiredoxins regulate oxidative protein folding and orchestrate the interplay between peroxidase and chaperone functions in the secretory pathway [16,109,111,129,130,131].

#### 3.4.5. Vitamin K Epoxide Reductase (VKOR)

**Figure 6:**
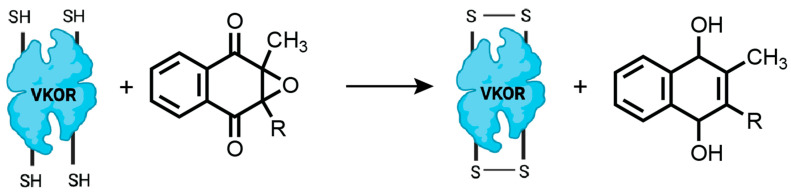
VKOR reduces vitamin K epoxide to hydroquinone.

Vitamin K epoxide reductase (VKOR) is an integral membrane enzyme in the endoplasmic reticulum that catalyzes the reduction of vitamin K epoxide to vitamin K hydroquinone, a critical cofactor for the post-translational γ-carboxylation of vitamin K-dependent proteins [120,132,133]. It serves as a significant source of oxidizing equivalents for PDI in the ER, especially when classical pathways such as ERO1 or PRDX4 are compromised or inactivated. Its ability to regenerate disulfide bonds in PDI ensures sustained oxidative folding capacity and redox balance within the ER, functioning as a parallel and compensatory mechanism alongside other ER oxidants [94,134]. VKOR plays a distinct role in oxidative protein folding within the endoplasmic reticulum (ER) by coupling quinone redox chemistry to the formation of disulfide bonds in proteins (Figure 6). Central to this catalytic cycle is the VKOR active site featuring a conserved CXXC motif, which cycles between reduced and oxidized states during electron transfer [120,132,133].

The oxidative folding pathway mediated by VKOR fundamentally depends on the unique redox properties of the vitamin K quinone ring. During its reaction cycle, VKOR reduces vitamin K epoxide to vitamin K quinone and then to vitamin K hydroquinone, a series of steps accompanied by the oxidation of the VKOR CXXC motif [134]. The oxidized CXXC motif forms a disulfide, which can subsequently be transferred to protein disulfide isomerase (PDI) family members via a cascade of thiol-disulfide exchange reactions. As a result, oxidizing equivalents originating from the vitamin K quinone ring are relayed through the sequential reduction of the quinone and oxidation of the CXXC motif to PDI, which then catalyzes disulfide bond formation in nascent proteins within the ER [120,133].

This mechanism has been convincingly demonstrated in microsomal systems where protein oxidation by VKOR was entirely dependent on the presence of PDI, with stable VKOR–PDI complexes shown by co-immunoprecipitation and by the ability of VKOR to transfer disulfides to several PDI family members [120]. Biochemical and structural studies further reveal that the electron flow is ultimately driven by the redox cycling of the quinone ring in VKOR, making it the terminal oxidant for this pathway. This distinguishes VKOR from other ER oxidases like ERO1, which use molecular oxygen as the terminal electron acceptor, as VKOR couples vitamin K metabolism and disulfide bond formation through quinone-mediated oxidoreductase chemistry [120,132].

Collectively, VKOR exemplifies how the quinone ring serves not only as a crucial cofactor for extracellular γ-carboxylation reactions but also as a central redox hub in the ER, channeling oxidative power through the CXXC motif to drive protein disulfide formation in partnership with PDI and related thiol isomerases. This quinone-dependent electron transfer system expands the ER’s oxidative folding capacity and connects vitamin K metabolism with the quality control of secreted protein maturation.

### 3.5. Chemical Oxidation of Thiol Isomerases

#### 3.5.1. Hydrogen Peroxide (H_2_O_2_)

Hydrogen peroxide serves as a key physiological oxidant of thiol isomerases, participating in both enzymatic and non-enzymatic oxidation pathways with reaction kinetics that are highly dependent on the protonation state of participating thiols [135,136]. Recent kinetic studies using full-length PDI have determined that the reaction of PDI’s redox-active cysteines (Cys53 and Cys397) with hydrogen peroxide proceeds with a second-order rate constant of 17.3 ± 1.3 M^−1^s^−1^ at pH 7.4 and 25 °C, approximately twice that determined for mutated PDI **a** domain alone, indicating that both PDI reactive thiols are oxidized with similar rate constants [137].

The reaction of H_2_O_2_ with PDI’s active site cysteines follows a multistep mechanism that is highly dependent on the protonation state of the participating thiols [76]. The nucleophilic cysteine (Cys53 in human PDI), with its lower pKa of approximately 6.0, exists primarily as a thiolate anion at physiological pH, making it particularly susceptible to oxidation by H_2_O_2_. The oxidation reaction proceeds through a nucleophilic attack by the thiolate on the peroxide oxygen, resulting in formation of a sulfenic acid intermediate (Cys-SOH) [138] (Equation (1)). This intermediate can follow one of two major pathways: in the presence of the resolving cysteine (Cys56), the sulfenic acid can condense to form a disulfide bond, effectively regenerating the enzyme’s active oxidized state. Alternatively, in the absence of an available resolving thiol or under conditions of oxidative stress, the sulfenic acid may undergo further oxidation to sulfinic (SO_2_H) (Equation (2)) or sulfonic (SO_3_H) acid (Equation (3)) derivatives, leading to irreversible enzyme inactivation [138,139,140]. These mechanisms are shown in the following reactions below:

-SH + H_2_O_2_ → SOH + H_2_O (1)

-SOH + H_2_O_2_ → SO_2_H + H_2_O (2)

-SO_2_H + H_2_O_2_ → SO_3_H + H_2_O (3)

The cellular context and availability of reducing equivalents largely determine which pathway predominates, with the ER’s relatively oxidizing environment favoring disulfide formation, while more extreme oxidative conditions promote overoxidation.

#### 3.5.2. Hypochlorous Acid (HOCl)

Hypochlorous acid (HOCl), generated by myeloperoxidase during inflammatory responses, represents a significantly more potent oxidant of thiol isomerases than H_2_O_2_ [141,142]. Proteomics studies using thiol-specific probes have identified PDI among the proteins particularly sensitive to oxidation by HOCl and model *N*-chloramines produced at inflammatory sites [143]. The reaction of HOCl with PDI’s active site cysteines occurs through several potential pathways, depending on the local microenvironment [141]. At physiological pH, HOCl primarily reacts with the thiolate form of cysteine to form a sulfenyl chloride intermediate (Cys-SCl), which is highly reactive and can subsequently participate in several reactions [138]. The sulfenyl chloride may react with a nearby thiol to form a disulfide bond, or it may hydrolyze to reform the sulfenic acid. Under conditions of high HOCl concentration, the sulfenyl chloride can undergo further chlorination to form stable sulfonamide derivatives or react with amine groups to form sulfonamides, both of which result in irreversible enzyme inactivation [138].

**Figure 7:**
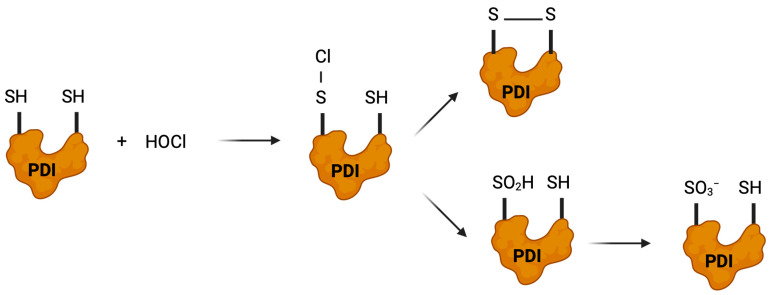
Mechanism of PDI oxidation by HOCl.

The biological consequences of HOCl-mediated oxidation are complex and context-dependent. At low, physiologically relevant concentrations, HOCl may enhance PDI’s oxidase activity by promoting rapid disulfide bond formation. (Figure 7). However, at the higher concentrations observed during chronic inflammation or phagocytic respiratory bursts, HOCl causes extensive oxidative damage to thiol isomerases (e.g., oxidation to further sulfur oxoforms), contributing to endothelial dysfunction and atherosclerosis [143,144,145,146,147]. The exquisite sensitivity of PDI’s active site cysteines to HOCl oxidation suggests an important role for this enzyme in sensing and responding to inflammatory oxidative stress.

#### 3.5.3. Reactive Nitrogen Species: ONOO^−^ and S-Nitrosation

Peroxynitrite (ONOO^−^), formed through the diffusion-limited reaction between nitric oxide (NO) and superoxide (O_2_^−^), represents one of the most physiologically relevant nitrosative stressors for thiol isomerases [148]. The reaction of ONOO^−^ with PDI occurs through multiple parallel pathways that depend on the protonation state of both the oxidant and the target cysteine [149]. At physiological pH, ONOO^−^ exists in equilibrium with its protonated form (ONOOH), with both species capable of oxidizing thiols but through distinct mechanisms [150,151,152]. Kinetic studies have demonstrated that the reaction of PDI’s redox-active cysteines with peroxynitrite is considerably faster than with hydrogen peroxide, with a second-order rate constant of (6.9 ± 0.2) × 10^4^ M^−1^s^−1^, and both Cys53 and Cys397 were kinetically indistinguishable [143]. Limited proteolysis, kinetic simulations, and mass spectrometry analyses confirm that peroxynitrite preferentially oxidizes the redox-active cysteine residues of PDI to corresponding sulfenic acids, which react with resolving thiols at active sites to produce disulfides [153]. This intermediate can then follow several potential fates: it may react with a nearby thiol to form a disulfide bond, undergo further oxidation to higher oxidation states, or react with NO to form an S-nitrosothiol (Cys-SNO) [154]. The formation of S-nitrosothiols on PDI represents a particularly important regulatory mechanism, as these modifications are reversible and function as redox switches to modulate enzyme activity during vascular inflammation and thrombosis. This is evidenced by nitrosothiol control of PDI function, with S-nitrosated PDI inhibiting platelet function and thrombosis while maintaining vascular quiescence [155]. The regulation involves both endothelial cells and platelets: NO scavenging results in exposure of free thiols and increased thiol isomerase activity on cell membranes, while exposure to NO^+^ carriers or elevation of endogenous NO levels results in S-nitrosation of PDI and decreased surface thiol reductase activity [155].

#### 3.5.4. Glutathione System and Thiol Redox Buffering

**Figure 8:**
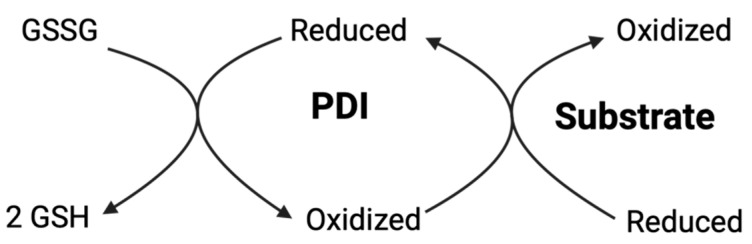
Mechanism of PDI oxidation by oxidized glutathione (GSSG).

The glutathione redox couple, consisting of reduced glutathione (GSH) and its oxidized form (GSSG), serves as the principal buffer controlling thiol-disulfide balance in the ER and other cellular compartments. Within the ER, the GSH:GSSG ratio is maintained at approximately 3:1, markedly more oxidizing than the cytosolic ratio of about 100:1, creating an environment uniquely suited for oxidative protein folding [6,156]. GSSG directly participates in the oxidative mechanisms of PDI by serving as a physiological disulfide donor, with reduced PDI being reoxidized by GSSG through thiol-disulfide exchange reactions that result in formation of a disulfide bond within the PDI active site and regeneration of two GSH molecules (Figure 8). Recent computational studies exploring the enzymatic mechanism of GSSG reduction by the reduced **a** domain of human PDI with atomistic resolution have revealed that the reaction proceeds in two stages: a thiol-disulfide exchange through nucleophilic attack of the Cys53-thiolate to the GSSG-disulfide followed by deprotonation of Cys56-thiol by Glu47-carboxylate, and a second thiol-disulfide exchange between the Cys56-thiolate and the mixed disulfide intermediate [157]. The calculated Gibbs activation energies of 18.7 kcal·mol^−1^ for the first stage and 7.2 kcal·mol^−1^ for the second stage are in excellent agreement with experimental barriers, demonstrating that PDI catalysis is mostly enthalpy-driven with minimal entropy changes [157]. The interplay between GSSG and PDI is further regulated by glutaredoxins, which can catalyze the reduction of PDI disulfides using GSH as a reductant, allowing for dynamic cycling between oxidized and reduced forms of PDI in response to changing cellular redox conditions.

#### 3.5.5. NADPH Oxidases (NOX) Derived Oxidative Species

NOX, especially NOX1, NOX2, and NOX4, represent critical local ROS sources that generate superoxide and hydrogen peroxide through regulated electron transfer from cytosolic NADPH to molecular oxygen [158,159]. The NOX2 isoform is particularly important in thrombosis and vascular biology [160], being rapidly assembled and activated at the plasma membrane of platelets and endothelial cells during vascular injury or inflammation. Upon activation, cytosolic subunits translocate to the membrane where they interact with the membrane-bound NOX2/p22phox complex, enabling electron transfer from NADPH through FAD and heme groups to molecular oxygen. The ROS generated by NOX enzymes, particularly H_2_O_2_, serve as potent oxidants for extracellular and membrane-associated proteins [161]. These proteins include thiol isomerases, such as PDI [162,163], with this oxidation facilitated by spatial colocalization of NOX isoforms and PDI on cell surfaces creating microdomains of high ROS concentration. Interestingly, the dehydrogenase region of NOX2 contains a cysteine-glycine-cysteine (CGC) triad that exhibits PDI-like disulfide reductase activity with recombinant NOX2 protein showing thiol-disulfide exchange capability. NOX2 and PDIA3 show some level of homology, suggesting additional complexity in NOX-PDI interactions beyond simple oxidant-target relationships [164].

#### 3.5.6. Dehydroascorbate (DHA)

**Figure 9:**
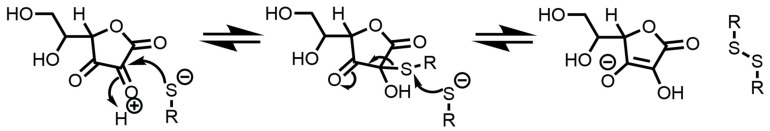
Mechanism of disulfide formation from oxidation of thiols by DHA. Adapted from [119].

DHA is a higher oxidation state of ascorbate formed through its action as an intracellular antioxidant, and has been proposed to participate in disulfide bond formation through interaction with PDI. Mechanistically, DHA can act as an electrophilic acceptor for reducing equivalents: the thiolate form of a cysteine residue can nucleophilically attack the oxidized carbon at position 2 or 3 of the DHA ring, transferring electrons from the thiol and resulting in the formation of ascorbate and a mixed disulfide intermediate [165]. This mechanism is shown in Figure 9. In the context of PDI, this reaction involves the nucleophilic cysteine in the reduced CXXC motif attacking DHA, which leads to the regeneration of ascorbate and formation of a disulfide bond within PDI’s active site.

Recent kinetic studies examining the possible role of PDI as a dehydroascorbate reductase have found the reaction too slow to be a major route for DHA reduction in the ER, with a second-order rate constant for the reaction of reduced PDI with DHA of only 12.5 M^−1^s^−1^ [165]. Rates of similar magnitude were obtained for other thioredoxin-superfamily members, though glutaredoxin was able to catalyze DHA reduction more rapidly through a monothiol mechanism. These findings suggest that while DHA can interact with PDI, it is unlikely to serve as a major physiological oxidant for thiol isomerases under normal cellular conditions.

Thus, while the chemical pathway exists by which DHA can accept electrons from PDI thiols and facilitate their oxidation resulting in ascorbate regeneration and disulfide formation. This route is kinetically and physiologically insignificant as a contributor to PDI oxidation in the endoplasmic reticulum under normal conditions. The prevailing evidence suggests that DHA-mediated PDI oxidation is largely overshadowed by faster, more efficient protein-based or small-molecule oxidants in the ER, such as ERO1, peroxidases, or GSSG [165].

#### 3.5.7. Molecular Oxygen (O_2_) in the Presence of Transition Metals (Iron and Copper)

**Figure 10:**
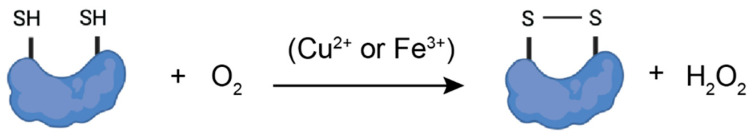
Dithiols are oxidized by transition metals forming disulfides with concomitant H_2_O_2_ generation.

Under physiological conditions, the direct oxidation of protein thiols by molecular oxygen is inefficient, as O_2_, being a ground-state diradical, reacts extremely slowly with thiol groups [166]. This is because the one-electron reduction potential of O_2_ is low, and thiols do not readily transfer electrons to O_2_ without catalysis [166]. However, when transition metals such as iron (Fe^2+^/Fe^3+^) or copper (Cu^+^/Cu^2+^) are present, they act as potent catalysts, dramatically increasing the rate of thiol oxidation and enabling the formation of disulfide bonds or other oxidation products (Figure 10).

Copper, in particular, is highly efficient in catalyzing thiol oxidation by O_2_. The mechanism typically involves formation of a transient Cu(II)-thiol complex, which facilitates electron transfer from the thiol to O_2_, generating thiyl radicals and ultimately forming disulfide bonds [167,168]. Detailed kinetic studies show that copper (II) ions increase the rate of aerobic thiol oxidation by several orders of magnitude compared to uncatalyzed reactions [169]. The reaction often proceeds via a thiyl radical intermediate: first, the thiol binds to Cu(II) forming a [Cu(II)-thiolate] complex; O_2_ then oxidizes the metal, allowing the release and coupling of two thiyl radicals to yield a disulfide. This effect is also seen in certain enzymes, such as the cuproenzymes superoxide dismutase and ceruloplasmin, which can exhibit thiol oxidase activities under defined conditions [168].

Iron, while abundant intracellularly, plays a more limited role in thiol oxidation under biological conditions, as it typically does not complex with thiols as efficiently as copper and is more commonly associated with the generation of reactive oxygen species (ROS) via Fenton chemistry [170]. In model systems, iron can enhance the oxidation of free thiols, but this process is less efficient than copper-mediated oxidation and is generally secondary to the pro-oxidant effects of copper in biological environments [166].

Overall, transition metals, especially copper, can enable O_2_ to oxidize protein and small-molecule thiols via catalytic mechanisms involving transient metal complexes and possible thiyl radical intermediates, significantly increasing the rate and prevalence of disulfide bond formation beyond what would occur with O_2_ alone. Iron can also play a role in thiol oxidation by generating superoxide which can in turn form disulfides.

### 3.6. Implications for the Oxidase Activity of Thiol Isomerases

The oxidase activity of PDI has been extensively studied in the context of the ER where it catalyzes disulfide bond formation in nascent proteins to ensure proper folding and maturation [7,97]. However, its extracellular oxidase function, particularly as it relates to endothelial cell function, thrombosis, and hemostasis, remains enigmatic and poorly defined. While PDI’s reductase activity is well-characterized in these vascular contexts, including the reduction of disulfides in integrins [51,52,53,54,55,56], GPIbα [57], and thrombospondin-1 [49,50], its oxidative substrates and mechanisms outside the ER are still largely unknown [24]. This knowledge gap limits our understanding of how PDI balances its redox functions in physiological and pathological states. In stark contrast, its oxidase activity where it introduces disulfide bonds into substrate proteins remains largely unexplored in vascular thrombotic disorders.

Pathological conditions such as sickle cell disease, diabetes, and cancer are characterized by ROS-mediated damage. Elevated ROS generation beyond the anti-oxidative defense mechanisms promotes a more oxidizing cellular microenvironment [135,171,172]. Under these conditions, PDI’s catalytic cysteines within its CXXC motifs may become persistently oxidized [24,41]. This oxidative shift could favor PDI’s oxidase activity over its reductase function. However, does oxidative stress merely inactivate PDI’s reductase capacity, or does it shift PDI toward a gain-of-function oxidase role? Does PDI catalyze pathological disulfide formation in key hemostatic elements, such as fibrinogen, vWF, or platelet receptors under oxidative conditions? Could this mechanism contribute to thrombus stabilization and propagation in diseases like diabetes and cancer [23]?

The study of PDI’s oxidase activity is hampered by significant technical challenges. Most available assays primarily assess reductase activity, including the di-eosin-GSSG reduction assay, which monitors PDI-mediated reduction of fluorescent glutathione disulfide [173], and the insulin turbidity assay [174]. The insulin turbidity assay tracks PDI-catalyzed reduction of insulin’s interchain disulfides, resulting in turbidity measurable by absorbance [174,175]. These assays are straightforward, accessible, and widely used [175]. In contrast, assays for oxidase activity are scarce and technically cumbersome. The scrambled RNase A refolding assay indirectly measures oxidase (and isomerase) activity by assessing the recovery of RNase function through PDI-mediated reformation of disulfides in denatured RNase; RNase activity is then quantified via cyclic cytidine 3′,5′-monophosphate (cCMP) hydrolysis [176,177,178]. However, this assay does not isolate oxidase activity, as it involves both reduction and oxidation steps and is highly dependent on the redox state of the scrRNase.

A more direct approach is the decapeptide oxidase assay, where PDI oxidizes cysteine residues in a synthetic peptide containing a tryptophan; disulfide bond formation quenches the intrinsic tryptophan fluorescence, measurable by fluorimetry [71,179,180]. This assay offers specificity but requires high-purity custom peptides and expensive instrumentation, limiting its practical utility. This assay is also highly dependent on the redox state of the peptide (e.g., oxidation of the peptide over time). These technical barriers have restricted efforts to identify PDI’s oxidative substrates in vascular biology and to delineate how its oxidase function contributes to thrombus formation and resolution.

Emerging methods hold promise for overcoming these challenges. Redox-sensitive biosensors (e.g., Reduction-Oxidation Green Fluorescent Protein; RoGFP [181]) could enable real-time monitoring of thiol isomerase oxidase activity in live cells, while microfluidic systems simulating blood flow may facilitate dynamic assays of disulfide bond formation in response to shear stress. Advancements in mass spectrometry-based approaches could identify thiol isomerase-dependent oxidative modifications in thrombi and endothelial cells, offering a systems-level view of its oxidative impact on vascular disorders.

While PDI’s reductase role in vascular biology is well established, its oxidase activity remains a critical and understudied frontier [51,52,53,54,55,56]. Given the oxidizing conditions prevalent in thrombotic and chronic inflammatory diseases, clarifying PDI’s oxidative substrates, regulatory mechanisms, and functional consequences is important. Such insights will not only advance our understanding of PDI’s dual roles in endothelial homeostasis and thrombus formation but may also inform the development of targeted interventions to modulate PDI’s redox balance for therapeutic benefit.

## 4. Natural Anti-Oxidative Galloylated Polyphenols Inhibit Thiol Isomerase Activity

### 4.1. Pharmacological Inhibitors of PDI

The inhibition of PDIs represents a powerful pharmacological approach owing to their role in oxidative protein folding, thrombosis, cancer, and infection [10,25,182,183]. PDIs catalyze disulfide bond formation and rearrangement in the endoplasmic reticulum; however, several mechanisms can lead to the extracellular localization of PDIs despite their C-terminal KDEL ER retention motif. These, including secretion by activated platelets and endothelial cells, increased ER stress causing saturation of retrieval pathways, and packaging into non-classical secretory vesicles [13,25,184,185,186,187,188].

Over the last decade, multiple classes of PDI inhibitors have been developed and characterized. Classic non-specific inhibitors include bacitracin (IC_50_ 20–1050 μM), which is known to inhibit viral entry and platelet accumulation, but lacks selectivity [10,183,189]. Modern irreversible inhibitors such as PACMA31 (IC_50_ of 10 µM in animal studies) covalently bind to the active-site cysteines of PDI and were shown to suppress tumor growth and thrombosis in preclinical models, with oral bioavailability and low toxicity in normal tissues [10,183,189,190]. Small-molecule antagonists like bepristat 2a selectively target the **b′** domain of PDIA1 and can prevent cancer cell adhesion and impair platelet aggregation [191,192]. Other selective molecules such as juniferdin (IC_50_ ~0.2–3 μM) inhibit both platelet aggregation and viral entry, while RB-11-ca and phenyl vinyl sulfonate derivatives are potent irreversible inhibitors with cytotoxic effects against cancer cell lines [10,189,193,194].

Natural products and dietary flavonoids, including quercetin-3-rutinoside (rutin, IC_50_ ~6 μM) and isoquercetin (IC_50_ ~9 μM), reversibly inhibit PDI and have advanced to clinical trial status as antithrombotics, demonstrating efficacy in reducing thrombin generation and platelet aggregation without cytotoxicity [10,25,189]. Other inhibitors such as cysteamine, DNTB, adenanthin, and E64FC26 have shown promise in targeting specific PDI family members or pathways, with some entering preclinical testing for cancer or inflammatory disease [189]. Antibody-based therapeutics, for example, those targeting AGR2 exert biological activity by directly suppressing tumor growth and metastasis in animal models [189,195].

Overall, the growing portfolio of pharmacological PDI inhibitors spans covalent active-site blockers, reversible domain-selective antagonists, dietary natural products, and monoclonal antibodies, each showing distinct profiles of potency, selectivity, and clinical applicability [10,25,189,193,194]. These advances lay the foundation for targeting PDIs in a spectrum of human diseases ranging from vascular thrombosis to cancer and viral infection, and underscore the expanding clinical significance of pharmacological thiol isomerase inhibition. 

### 4.2. Galloylated Polyphenols as Thiol Protective Antioxidants

Galloylated polyphenols are plant-derived compounds characterized by one or more galloyl (trihydroxybenzoyl) groups, which endow these molecules with potent redox activity and antioxidant capacity [196]. Classic examples include tannic acid, epigallocatechin gallate (EGCG), pentagalloylglucose (PGG), punicalagin, and galloylated theaflavins [32]. These polyphenols can scavenge reactive oxygen species and chelate redox-active metal ions, thereby protecting cellular thiols from oxidative stress [197]. In plants, galloylated polyphenols are thought to have evolved as defensive antioxidants to safeguard proteins, many of which contain critical cysteine thiols, against oxidative stress and pathogens [198]. Recently, a study highlighted the antimicrobial, anti-inflammatory, and antioxidant activity of the galloylated glucose derivative, 1-O-galloyl-6-O-p-coumaroyl-d-glucose, derived from the medicinal Omani plant *Anogeissus dhofarica* [199]. Notably, mammals cannot synthesize galloyl moieties; they are instead acquired through dietary means as they are present in foods and beverages such as tea, coffee, pomegranate, grapes, and chocolate products [200]. Epidemiological studies have linked high consumption of polyphenol-rich teas and coffees with improved cardiovascular outcomes, indicating a reduced risk of thrombotic events [201]. While multiple factors likely contribute to this result, galloylated polyphenols’ antioxidant properties are believed to play a role [32]. By maintaining an environment favoring chemical reduction, these compounds can help preserve the function of thiol-dependent proteins in the vasculature, preventing deleterious thiol oxidation which may trigger platelet activation or coagulation [202]. In essence, galloylated polyphenols act as natural guardians of thiol groups, curbing oxidative modifications that would otherwise promote thrombogenic protein misfolding or activity [12].

The concept of targeting thiol isomerases for antithrombotic therapy is not entirely new and has been developing for over a decade. However, the identification of galloylated polyphenols as a potential approach validates and extends prior research. Quercetin-3-rutinoside (rutin) inhibits PDI and can block thrombus formation in vivo, proposing the idea that small molecules could drug these enzymes and achieve antithrombotic effects [25]. Subsequent studies detailed how rutin and its analogues bind to an allosteric pocket on PDI, the **b′x** domain interface at His256, and prevent PDI from amplifying prothrombotic activity [26,203]. The current findings on galloylated polyphenols align with this trajectory but introduce key advances. A crucial finding from the antithrombotic experiments was that mice treated with galloylated polyphenols did not exhibit prolonged bleeding times [32,64]. This suggests a degree of specificity for thrombosis over hemostasis, which could serve as a monumental advantage. Prior PDI inhibitors like rutin also showed minimal bleeding risk in animal models, so this aligns with the notion that inhibiting thiol isomerases uncouples thrombosis from hemostasis [204]. However, the long-term safety of polyphenol supplementation remains to be fully evaluated. Off-target effects need consideration as these compounds often have multiple targets. For example, chronic inhibition of MAPKs or PKC by dietary polyphenols could have beneficial anti-inflammatory effects but could also modulate immune responses in unforeseen ways [205]. Fortunately, galloylated polyphenols have been consumed by humans for centuries in foods, with a generally favorable safety profile [206,207]. The current work challenges potential skepticism about their biomedical relevance by providing tangible evidence of efficacy in disease models.

### 4.3. COVID-19-Associated Coagulopathy and Redox Enzymes

Severe COVID-19 infection is frequently accompanied by profound coagulopathy. Patients with COVID-19 exhibit widespread microvascular thrombosis, elevated D-dimer levels, and disseminated intravascular coagulation in the worst cases [208,209]. Autopsy studies of COVID-19 victims have revealed extensive pulmonary endothelial inflammation and fibrin-rich microthrombi in the lung vasculature [210,211]. This prothrombotic state, termed COVID-19-associated coagulopathy, arises from multiple converging mechanisms [212]. Hyperinflammatory responses, such as cytokine storms, trigger tissue factor expression, activate coagulation, cause endothelial cell injury, and promote platelet hyperreactivity, thereby driving thrombosis in this condition [213]. Notably, an oxidative stress component is also evident in COVID-19, as massive inflammation generates ROS that can oxidize critical vascular thiols and dysregulate redox-sensitive clotting pathways [214]. PDI and its family member oxidoreductases have garnered attention in this context: these enzymes are secreted during vascular injury and facilitate thiol-disulfide exchange reactions that activate platelets and coagulation proteins [215]. Excessive oxidative stress in COVID-19 may excessively express or dysregulate these thiol isomerases, compounding the thrombotic risk. Indeed, elevated circulating PDI has been associated with thrombotic complications in other inflammatory diseases [216] and could play a similar pathophysiological role in COVID-19. Recognizing that COVID-19 uniquely links a viral infection with a high incidence of thrombosis, researchers have been seeking therapeutic strategies that can address both the viral replication and the coagulopathic aspects of the disease. One intriguing approach is to target common molecular denominators of both processes such as redox-active cysteine enzymes to achieve dual antiviral and antithrombotic effects.

Extracellular PDI has emerged as a central driver of COVID-19 immunothrombosis. It was shown that activated platelets and endothelial cells secrete PDI, which catalyze disulfide rearrangements in tissue factor (TF) and sharply accelerate fibrin generation in both acute and “long COVID” settings [217,218]. Building on this, PDIA1 on the platelet surface governs the shedding of procoagulant extracellular vesicles (pEVs) independently of aggregation or Ca^2+^ signaling [219]. PDIA1 inhibition markedly reduces pEV release which likely contributes to thrombus formation in severe COVID-19 [219]. Complementarily, it was identified via molecular docking studies and human plasma assays that the flavonoid naringin both potently inhibits PDI and delays clotting by this PDI blockade [220]. Finally, a review of sepsis and COVID-19-associated coagulopathies highlights PDI-mediated thiol-disulfide exchange on TF and platelet surfaces as a unifying procoagulant mechanism across both diseases [221]. Together, these findings suggest that galloylated polyphenols which bind to and block PDI’s active site thioredoxin motif could simultaneously prevent coagulation and pEV production, thereby impeding multiple pathways that contribute to the COVID-19 immunothrombosis signaling cascade.

### 4.4. Discovery of Galloylated Polyphenols as Dual Inhibitors of Viral Protease and Thiol Isomerases

Early in the pandemic, the essential SARS-CoV-2 main protease (Mpro, also called 3CLpro) was identified as a prime antiviral drug target [222]. Mpro is a cysteine protease that uses an active-site cysteine (Cys145) and histidine (His41) dyad to cleave viral polyproteins [223]. Interestingly, this cysteine-dependent enzyme resembles thiol isomerases like PDI that also rely on catalytic cysteine-histidine motifs [32]. The similarity of a viral cysteine protease with host cysteine isomerases in COVID-19 pathology raised the question: could a single compound class inhibit both the virus and PDI-related thrombosis? To explore this, a study conducted a high-throughput screen of 1019 structurally diverse flavonoids for inhibitors of SARS-CoV-2 Mpro [224]. Strikingly, several of the top hits emerging from this screen were galloylated polyphenols. One particular compound, pinocembrin 7-O-(3″-galloyl-4″,6″-(S)-hexahydroxydiphenoyl)-β-D-glucose (abbreviated PGHG), stood out [224]. PGHG is a complex flavonoid glucoside bearing both a galloyl group and a hexahydroxydiphenoyl moiety [32]. It was found to potently inhibit Mpro activity in vitro (IC_50_ 6.5 µM) and to suppress SARS-CoV-2 replication in cell culture [224]. PGHG’s galloyl-rich structure may suggest redox reactivity hinting at interaction with cysteine enzymes in general. Subsequent testing confirmed that PGHG does have a dual mode of action: it not only blocks the viral protease but also inhibits several PDI family members [32,224]. Additionally, multiple other polyphenols including proanthocyanidin and the galloylated punicalagin have demonstrated similar PDI antagonism.

**Figure 11:**
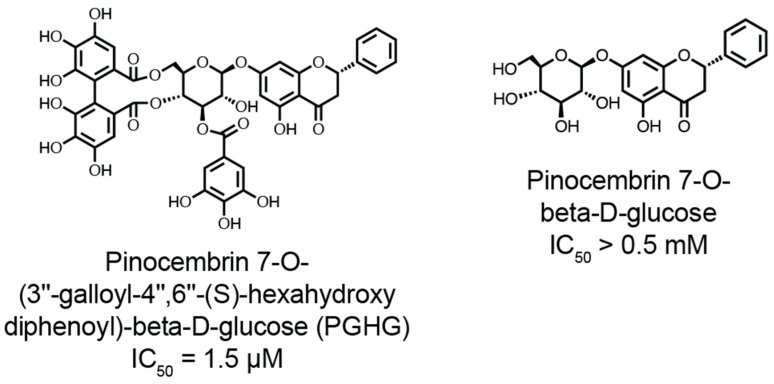
Pinocembrin 7-O-(3″-galloyl-4″,6″-(S)-hexahydroxydiphenoyl)-beta-D-glucose (PGHG) antagonizes the reductase activity of PDI at low μM relative to its pinocembrin analog control. IC_50_ profiles reported from [32].

Follow-up studies demonstrated that PGHG broadly antagonizes the thiol-disulfide reductase activity of PDI and its family members ERp57, ERp5, ERp72, and ERp46 [32]. These enzymes are important for platelet function and fibrin generation [64]. In functional assays, PGHG dose-dependently inhibited PDI’s ability to reduce disulfide bonds in dieosinediglutathione and insulin with IC_50_s in the low micromolar range [32] (Figure 11). This inhibition crucially translated into an antithrombotic effect in vivo. In mouse models of arterial thrombosis, administration of PGHG or related galloylated polyphenols significantly delayed thrombus formation without causing excessive bleeding. For example, oral punicalagin prolonged carotid artery occlusion times in mice, and about half of arteries never occluded under its influence, yet tail-bleeding times and blood loss were unchanged compared to controls [32]. This suggests that targeting thiol isomerases can inhibit pathological thrombosis while sparing normal hemostasis.’

The discovery that galloylated polyphenols concurrently inhibit a coronavirus cysteine protease and host thiol isomerases underscores a remarkable example of “dual pharmacology.” It appears that the same chemical features that allow these polyphenols to interfere with one cysteine-dependent enzyme, Mpro, also enable them to bind and inhibit others in the PDI family. Multiple members of this compound class were found to have parallel antiviral and antithrombotic activities. For instance, theaflavin-3,3′-digallate, a black tea polyphenol, was among the top nine Mpro inhibitors in the flavonoid screen and later demonstrated PDI inhibition. Likewise, tannic acid and pentagalloylglucose (PGG), both galloylated tannins, have been reported to inhibit SARS-CoV-2 Mpro (IC_50_ ~13 μM for tannic acid) and to block SARS-CoV-2 infection in vivo. Punicalagin, an ellagitannin from pomegranate, was also highlighted for its dual action: it inhibits the SARS-CoV-2 3CL protease and has demonstrated inhibition of SARS-CoV-2, including the Omicron variant, infection in vitro [224]. Recent studies reported that punicalagin and related tannins can prevent viral entry into cells and replication, in part by binding viral proteins, which complements their antithrombotic potential [225]. The same galloylated polyphenols inhibit Mpro (IC_50_ ~5 μM) and block PDI-mediated disulfide reduction in vitro [32,224]. By simultaneously targeting viral replication and pathological clotting, galloylated polyphenols represent a unique therapeutic avenue for COVID-19 and other diseases characterized by both processes. Nevertheless, their clinical translation hinges on overcoming poor oral bioavailability and extensive metabolism. Future efforts should therefore focus on optimized delivery to fully harness their dual antiviral and antithrombotic potential.

### 4.5. Targeting the Catalytic CXXC Motif of Thiol Isomerases

Emerging evidence indicates galloylated polyphenols inhibit thiol-disulfide oxidoreductases by directly engaging the enzymes’ catalytic CXXC motif. PDI and its family members contain one or more active sites characterized by the Cys-X-X-Cys (CXXC) motif within a thioredoxin-like domain [226]. In the oxidized state, these two cysteines form a disulfide bond, and in the reduced state, a nucleophilic thiolate on the *N*-terminal cysteine can attack substrate disulfides. Crucially, a conserved histidine adjacent to the catalytic cysteine (typically in a CGHC sequence, where Histidine is the third residue) helps polarize and deprotonate the cysteine thiol, facilitating its reactivity. This Cys-His relationship in the active site is analogous to the catalytic Cys-His dyad of SARS-CoV-2 Mpro, which galloyl polyphenols may be able to exploit [224].

PGHG inhibits the isolated **a** and **a′** domains of PDI (each containing a CGHC motif) as effectively as it inhibits full-length PDI, indicating that the compound acts at the catalytic domains themselves [32]. Moreover, PGHG had little effect on PDI fragments lacking the active-site cysteines (the **b** or **b′** domains), and it did not inhibit a mutant PDI in which both active-site cysteine pairs were replaced, reinforcing that the native CXXC motifs are required for its action [32]. Early hypotheses posited that galloylated polyphenols might covalently modify the catalytic cysteines by, for instance, Michael addition or oxidative conjugation given their polyphenolic reactivity. However, careful biochemical analyses did not support a covalent mechanism. PGHG did not block the alkylation of PDI’s free thiols by maleimide probes, nor did it induce any detectable shift in PDI’s molecular weight that would signal adduct formation [32]. Instead, reversibility tests showed that PDI activity recovered fully upon dilution of PGHG, unlike the irreversible inhibition seen with *N*-ethylmaleimide. These results indicate that PGHG inhibits PDI through a non-covalent, reversible interaction at the enzyme’s active site [32].

**Figure 12:**
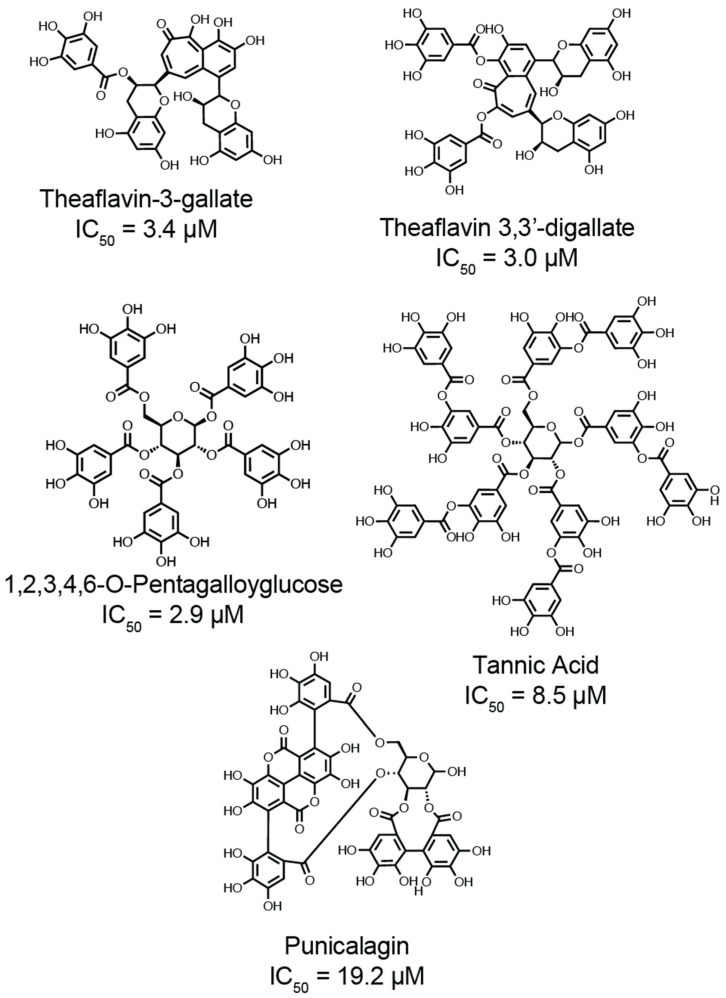
Galloylated polyphenols of the theaflavin, gallotannins, and ellagitannin showing potency against PDI reductase activity. IC_50_ profiles published from [32].

Molecular docking and mutagenesis have pinpointed the nature of this interaction. Computational modeling of PGHG bound to PDI’s **a** domain revealed that one of PGHG’s galloyl subunits fits into a pocket near the CGHC motif, forming hydrogen bonds with the side chain of the active site histidine, His55 [32]. Consistent with this model, mutation of that histidine and the equivalent His399 in the **a′** domain to alanine, aspartate, or arginine rendered PDI largely resistant to PGHG inhibition [32]. In contrast, those mutations did not affect inhibition by quercetin-3-rutinoside (rutin), a flavonoid PDI inhibitor known to bind a different site. Thus, PGHG and related galloylated polyphenols represent a distinct mechanistic class: they target the thioredoxin active-site cleft of thiol isomerases, relying on the presence of the CXXC motif and especially the catalytic histidine for binding [32]. This mechanism is subtly different from that of smaller flavonoids like rutin or quercetin, which have been shown by NMR to bind an adjacent allosteric pocket on PDI (the **b′** domain hydrophobic site at His256) rather than directly at the active site [26,203]. The galloylated polyphenols, by virtue of their larger size and multiple phenolic groups, appear capable of spanning and bridging the active-site region, perhaps transiently occupying the substrate-binding cleft of PDI to block its reductase function. The galloylated polyphenol compounds tested against PDI reductase activity [32] are shown in Figure 12.

Crucially, this inhibition focused at the active site extends well beyond PDI itself. Every family member bearing the canonical CGHC motif, such as PDI, ERp57, ERp5, ERp72 and ERp46, is susceptible to galloylated polyphenols antagonism with similar potency, reflecting their shared thioredoxin fold and conserved active-site architecture. By homing in on the universally conserved catalytic machinery of thiol isomerases, galloylated polyphenols act as broad-spectrum inhibitors capable of simultaneously dampening the prothrombotic contributions of multiple enzymes released at sites of vascular injury. Since PDI supports tissue factor activation and platelet fibrin generation, while ERp5 and ERp57 regulate platelet receptor function, their collective inhibition likely underlies the robust antithrombotic efficacy observed in vivo which is exemplified by punicalagin’s ability to delay arterial occlusion without impairing hemostasis [32]. In short, galloylated polyphenols exert their antithrombotic action by binding to thiol isomerase active sites, shielding the critical CXXC thiols from catalysis or hyperoxidation, and thus preserve redox balance in a way that blunts thrombogenic pathways.

Unlike smaller flavonoids such as rutin, which bind an allosteric hydrophobic pocket, galloylated polyphenols engage with the active site directly via the CGHC motif. This discovery upends the assumption that PDI’s catalytic cleft is inaccessible to small molecules, revealing that larger, multi-phenolic structures can span and block the thioredoxin active site cleft itself. Yet despite supportive docking and mutagenesis data, definitive proof of this binding position remains elusive: high-resolution X-ray crystallography or NMR of a PDI-polyphenol complex is needed to confirm the precise interactions, determine whether inhibition is purely competitive or includes allosteric effects, and guide rational optimization of these dual-action inhibitors.

## 5. Conclusions

Thiol isomerases constitute a 21-family member of enzymes that function to make and break disulfides and are important for thrombus formation. This mechanism is through the reductase, oxidase, and isomerase catalytic functions. They could be regulated enzymatically by other oxidases or through chemical oxidation, influencing their efficiency in transferring disulfides to protein substrates. Thiol isomerases are antagonized by anti-oxidative galloylated polyphenols. This antagonism could be useful in dual pharmacology in settings where coagulopathy is associated with oxidative stress.

## 6. Patents

M.Y. has a patent pending with Robert Flaumenhaft at Beth Israel Deaconess Medical Center entitled, “Compounds and Methods for Using Galloylated Polyphenols to Treat Diseases Mediated by Thiol Isomerases” that is associated with the highlighted galloylated polyphenol studies in this review.

## Figures and Tables

**Table 1:** Human thiol isomerase family members.

Thiol Isomerase	Gene Name	Amino Acids	Molecular Mass (kD)	Domain Structure	CXXC Motif
PDI	*P4HB*	508	57.1	a-b-b′-a′	_53_CGHC_56_; _397_CGHC_400_
PDIp	*PDIA2*	525	58.2	a-b-b′-a′	_71_CGHC_74_; _418_CTHC_421_
PDIr	*PDIA5*	519	59.5	a^0^-a-b-b′	_182_CSMC_185_; _305_CGHC_308_; _426_CPHC_429_
PDILT	*PDILT*	584	66.6	a^0^-a-b-b′	_72_SKQS_75_; _417_SKKC_420_
ERp5	*PDIA6*	440	48.1	a-a-b	_55_CGHC_58_; _190_CGHC_193_
ERp18	*TXNDC12*	172	19.2	a	_66_CGAC_69_
ERp27	*ERP27*	273	30.5	b-b′	----
ERp29	*ERP29*	261	29	b-b′	----
ERp44	*ERP44*	406	46.9	a-b-b′	_58_CRFS_60_
ERp46	*TXNDC5*	432	47.6	a-b-b′	_89_CGHC_92_; _217_CGHC_220_; _350_CGHC_353_
ERp57	*PDIA3*	505	56.8	a-b-b′-a′	_57_CGHC_60_; _406_CGHC_409_
ERp72	*PDIA4*	645	72.9	a^0^-a-b-b′-a	_91_CGHC_94_; _206_CGHC_209_; _555_CGHC_558_
ERp90	*TXNDC16*	825	93.5	a-a-a-b-b′	_84_CX_8_C_93_; _216_CX_9_C_226_; _449_CX_6_C_456_
ERdj5	*DNAJC10*	793	91.1	a^0^-b-a-a-a	_158_CSHC_161_; _480_CPPC_483_; _588_CHPC_591_; _700_CGPC_703_
TMX1	*TMX1*	280	31.7	a	_56_CPAC_59_
TMX2	*TMX2*	296	34	b	_167_SNDC_170_
TMX3	*TMX3*	454	51.8	a-b-b′	_53_CGHC_56_
TMX4	*TMX4*	349	38.9	a	_64_CPSC_67_
TMX5	*TXNDC15*	360	39.6	b′	_220_CRFS_223_
AGR2	*AGR2*	175	19.9	a	_81_CPHS_84_
AGR3	*AGR3*	166	19.1	a	_71_CQYS_74_

## Data Availability

No new data were created or analyzed by this study. Data sharing is not applicable to this article.
